# Long noncoding RNA PANDAR blocks *CDKN1A* gene transcription by competitive interaction with p53 protein in gastric cancer

**DOI:** 10.1038/s41419-017-0246-6

**Published:** 2018-02-07

**Authors:** Jun Liu, Qiwen Ben, Eryi Lu, Xiangyi He, Xiaoqun Yang, Jun Ma, Wen Zhang, Zhiming Wang, Tianshu Liu, Jianjun Zhang, Hongxia Wang

**Affiliations:** 1 Department of Oncology, Shanghai General Hospital, Shanghai Jiao Tong University School of Medicine, 200080 Shanghai, PR China; 2 Department of Gastroenterology, Ruijin Hospital, Shanghai Jiao Tong University School of Medicine, 200025 Shanghai, PR China; 3 Department of Stomatology, Renji Hospital, Shanghai Jiao Tong University School of Medicine, 200127 Shanghai, PR China; 4 Department of Pathology, Fudan University Shanghai Cancer Centre, 200032 Shanghai, PR China; 5 Department of Integrative Medicine, Zhongshan Hospital, Fudan University, 200032 Shanghai, PR China; 6 Department of Oncology, Zhongshan Hospital, Fudan University, 200032 Shanghai, PR China; 7 Department of Oral and Maxillofacial-Head and Neck Oncology, Ninth People’s Hospital, Shanghai Jiao Tong University School of Medicine, 200011 Shanghai, PR China

## Abstract

Emerging evidence indicates that lncRNAs play important roles in cancer tumourigenesis and could be used as potential diagnostic biomarkers or therapeutic targets. However, the clinical significance and molecular mechanism of lncRNAs in gastric cancer (GC) is still unclear. The aim of this study was to explore the expression and role of lncRNAs in GC. The relative expression level of lncRNAs in GC samples was examined by an lncRNA microarray analysis, northern blot analysis and qRT-PCR analysis. A Kaplan−Meier survival analysis and univariate and multivariate Cox proportional hazards models were performed to evaluate the clinical and prognostic significance of PANDAR (promoter of CDKN1A antisense DNA damage activated RNA) in GC patients. The binding activity of PANDAR with the p53 protein was analysed by an RNA immunoprecipitation analysis and RNA pull-down analysis. The depletion of PANDAR was conducted using the CRISPR/Cas9 system for PANDAR. The biological functions of PANDAR in GC cells were determined both in vitro and in vivo. Upregulated PANDAR in GC patients was positively correlated with increased tumour size, advanced TNM classification and a poor survival rate in GC patients. The ROC curves identified that the PANDAR level was a marker for discriminating the early-stage tumour group from the healthy group, the metastasis group from the non-metastasis group and the chemoresistance group from the chemosensitive group in GC patients. As a target, the *CDKN1A* gene was successfully downregulated by PANDAR. PANDAR controlled the transcription of the *CDKN1A* gene by competitively binding with p53 protein. In combination with a p53 activator (nutlin3), the knockout of PANDAR by CRISPR/Cas9 technology synergistically inhibited GC tumour growth in vivo. Our results suggest that the PANDAR is a powerful diagnostic and therapeutic marker for patients with GC and, combined with other chemotherapeutics, may have distinct antitumour effects.

## Introduction

Gastric cancer (GC) is one of the most common cancers and accounts for a notable proportion of global cancer mortality^1^. Gastric cancer is often diagnosed at an advanced stage in the majority of patients and is associated with poor 5-year survival rates^2^. Despite recent advances in medical treatment, there has been little improvement in the early diagnosis and treatment of GC^3,4^. Increasing evidence indicates that long noncoding RNAs (lncRNAs) play critical roles in a wide range of biological processes, including cell development, differentiation, immune responses and tumourigenesis^5,6^. Understanding the contributions of lncRNAs to GC progression will offer insights into tumour transformation and help to identify new biomarkers and novel treatment targets for this disease.

LncRNAs refer to RNAs that lack coding potential, are >200 bp in length and are pervasively transcribed in the genome^7^. LncRNAs play critical roles as tumour suppressors or oncogenes by activating or silencing the expression of protein-coding genes^8^. The lncRNA HOTAIR induces the genome-wide re-targeting of Polycomb Repressive Complex 2 and leads to altered histone H3 lysine 27 methylation and increased breast cancer metastasis^9–11^. The lncRNA MALAT1 is required for G1/S and mitotic progression, and p53 is a major downstream mediator of MALAT1 activity^12^. For GC, the upregulation of HOTAIR is associated with more venous invasion, frequent lymph node metastases and a lower overall survival rate^13^. The overexpression of the lncRNA H19 dramatically promotes GC cell proliferation and metastasis by the direct upregulation of the ISM1 protein^14^. The lncRNA GAPLINC is highly expressed in GC tissues and mainly changes the migratory ability of the cancer cells by altering the levels of CD44 mRNA^15^. Low lncRNA GAS5 expression in GC is associated with poorer overall survival, and the ectopic expression of GAS5 influences GC cell proliferation via regulating E2F1 and *CDKN1A* expression^16^. Acting as a competing endogenous RNA, lncRNA-FER1L4 regulates the expression of PTEN, CDKN1A and E2F1 through its miRNA response elements to compete for miR-106a-5p^17^. The lncRNA LEIGC suppresses tumour growth and enhances the sensitivity of GC cells to 5-fluorouracil by inhibiting the epithelial-to-mesenchymal transition^18^. Therefore, deeply understanding the roles of lncRNAs in tumourigenesis has an important significance in the development of molecular-targeted therapy.

In this study, we report findings implicating the lncRNA PANDAR (promoter of CDKN1A antisense DNA damage activated RNA) (LNCipedia.org; Gene ID: PANDAR) in GC based on the use of global microarray analyses in human GC specimens. We analysed the relationship between PANDAR levels and the clinicopathological features of GC, including clinical outcome. We deeply investigated the biological effect and mechanisms of altered PANDAR levels on the phenotypes of GC cells in vitro and in vivo. Our findings suggest that PANDAR may represent a novel indicator of poor prognosis in GC and may be a potential diagnostic and therapeutic marker.

## Materials and methods

### Patients and reagents

Between April 2007 and April 2008, we recruited 146 patients with primary GC from the Zhongshan hospital and Shanghai Cancer Center, Fudan University. All the patients (excluding the stage IV disease) underwent an R0 resection (with no tumour cells at the margin) with D2 lymph node dissection, and all the patients histopathologically confirmed for the diagnosis of stages II and III received 6–8 courses of chemotherapy-based fluorouracil following the operation. The Ethics Committee of Fudan University approved our study. All the participants provided written informed consent prior to enrolment. The human GC cell lines were obtained from the American Type Culture Collection (Manassas, VA, USA) and were identified through an STR analysis. The p53 antibody (#9282) and p21 antibody (#8831) were purchased from Cell Signaling Technology (Beverly, MA, USA). Nutlin-3 (S1061) was purchased from Selleck (Princeton, NJ, USA).

### CRISPR/Cas9-mediated deletion of PANDAR/TP53/CDKN1A

The CRISPR (clustered regularly interspaced short palindromic repeats)/Cas9 system has the ability to infect a broad variety of mammalian cells by co-expressing a mammalian codon-optimized Cas9 nuclease along with a single guide RNA (sgRNA) to facilitate genome editing. The lentiCRISPRv2 (Plasmid #52961)) was obtained from Addgene (Cambridge, MA, USA). The lentiCRISPRv2 plasmid contains two expression cassettes, hSpCas9 and the chimeric guide RNA. The vector was first digested using BsmBI (#R0580S, NEB, Beijing, China), and a pair of annealed oligos was cloned into the single guide RNA scaffold. The oligos were designed based on the target site sequence and were flanked on the 3′ end by a 3 bp NGG PAM sequence. The sgRNA sequence for PANDAR was designed using the CCTop-CRISPR/Cas9 target online predictor (http://crispr.cos.uni-heidelberg.de/index.html). The sgRNA sequences for TP53 and CDKN1A were designed using E-CRISP-Version 5.2 website (http://www.e-crisp.org/E-CRISP/). After 72 h of infection with the lentiCRISPRv2 plasmid, the single cell clones were selected by puromycin at a concentration of 10 μg/ml.

### LncRNA microarray analysis

The lncRNA expression profiles were investigated using the Agilent Human lncRNA array 8*60K (OE Biotech, Shanghai, China). The RNA samples were first reverse transcribed into cDNA, and these cDNA samples were then labelled using the Low Input Quick-Amp Labeling Kit (Agilent Technologies, Santa Clara, CA, USA). The labelled cDNA samples were used as probes to hybridize to the lncRNA microarrays. After the samples were hybridized, the microarrays were scanned with an Agilent microarray scanner. Feature Extraction software (version 10.7.1.1, Agilent Technologies) was used to analyse the array images to obtain the raw data. Gene-Spring software (version 12.5, Agilent Technologies) was employed to finish the basic analysis of the raw data. Initially, the raw data were normalized using the quantile algorithm. The probes with at least 100% of the samples in any one condition out of two conditions having flags that indicated ‘Detected’ were chosen for further data analysis. The differentially expressed lncRNAs were then identified through the fold change, and *p* values were calculated using *t*-tests. The thresholds set for the up- and downregulated genes were a fold change ≥2.0 and a *p* value ≤ 0.05.

These microarray data were approved and were assigned the GEO accession number GSE84787.

### Northern blot analysis

PANDAR levels were measured by northern blotting using an Ambion Northern Max-Gly Kit (Austin, TX, USA). The total RNA was electrophoresed on a 1% agarose gel and was siphoned to a positively charged nylon membrane (NC). The RNA was then fixed to the NC membrane using UV crosslinking. The cross-linked membrane was prehybridized with ultrahyb-oligo hybridization buffer and hybridized with the PANDAR-specific oligonucleotide probes (AACATTGGGTGGGGCGAGTCAT) labelled with digoxigenin-ddUTP in roller bottles. The probe was designed using the EXIQON website (https://www.exiqon.com).

### Plasmid construction

Full-length sequences of PANDAR and the wild-type *TP53* gene were amplified by PCR. AGS cells were used as the PCR template. The PCR products were first sub-cloned into the T vector and were then cloned into the pLKO.1 plasmid (Addgene, Cambridge, MA, USA). We used the Phusion Site-Directed Mutagenesis Kit (Life Technologies, Grand Island, NY, USA) to construct the *TP53* mutated vector. For lentiviral vector packaging, three plasmids are transfected into 293T cells, including the transfer vector (3 μg), delta 8.9 (3 μg) and VSV-G (1 μg). After a media change overnight, the supernatant containing the virus was stored.

### QRT-PCR analysis

Total RNA was extracted from GC tissues and cell lines using the TRIzol Reagent (Invitrogen, Shanghai, China). For the reverse transcript reaction, 1 μg of total RNA was used and was mixed with the random primer. The reaction was carried out for 30 min at 16 °C, 30 min at 42 °C, and 5 min at 85 °C and was then held at 4 °C. QRT-PCR was performed to measure PANDAR expression levels using the PowerUp™ SYBR^®^ Green Master Mix (Life Technologies, Grand Island, NY, USA). The reaction conditions were as follows: 1 cycle of 95 °C for 1 m; 38 cycles of 95 °C for 5 s and 65 °C for 30 s. We used the delta delta CT method to calculate the fold change in gene expression between the different groups. The primers used for these analyses are listed in Supplementary Table 4.

### Immunoblotting analysis

The whole cell lysates (50 μg) were electrophoresed using 10% polyacrylamide gels and were transferred to a PVDF membrane. The membrane was incubated with the p53 antibody (1:1000), p21 antibody (1:1000) or HA mouse antibody (1:3000). The secondary antibodies (1:3000) were labelled with horseradish peroxidase (HRP). The signals were checked using an autoradiography film when the HRP substrate was added to the membrane.

### ChIP analysis

The CHIP protocol was described in detail in a previous study^19^. The proteins were cross-linked to the DNA by adding formaldehyde at room temperature for 10 min, and the DNA was sheared to an average fragment size of 300−500 bp by sonication. Subsequently, immunoprecipitation was performed using the p53 antibody. The purified DNA was quantified by qRT-PCR using the PowerUp™ SYBR^®^ Green Master Mix (Life Technologies, Grand Island, NY, USA). The primer pairs used for analysis are described in Supplementary Table 4.

### RNA immunoprecipitation (RIP) analysis

GC cells were treated with the appropriate vectors for 48 h and were harvested by trypsinization. The nuclei were pelleted by centrifugation, were re-suspended using Magna Nuclear RIP™ Kits (Millipore, Billerica, MA, USA) and were mechanically sheared using a homogenizer. The HA antibody was added to the nucleic extraction, and it was incubated overnight at 4 °C. The HA beads were then added and incubated for 4 h at 4 °C. The HA beads were pelleted, washed and re-suspended in 1 ml of TRIzol. The isolated RNA was reverse transcribed to cDNA and then analysed with qRT-PCR.

### RNA pull-down analysis

The RNA pull-down analysis protocol was described in detail in a previous study^20^. The biotinylated PANDAR and U6 RNA were mixed with proteins from the nuclear extract of the cancer cells. The complex of the biotinylated PANDAR and proteins was purified using streptavidin-agarose. The proteins were then eluted from the RNA-protein complex and detected by immunoblotting using the p53 antibody or HA antibody.

### Cell proliferation and clone formation analysis

AGS cells and SNU-1 cells were transfected with the lenti-PANDAR vector or the knockout of PANDAR for 48 h and were seeded onto 96-well plates (1000 cells/per well) and 10 cm dishes (1000 cells/per well). Cell viability was measured daily using the CCK-8 assay (Dojindo, Shanghai, China). The clone formation ability was determined after 2 weeks of culture by crystal violet staining.

### Immunohistochemistry analysis

The mouse tumour tissues were fixed in formalin, embedded in paraffin, spliced into 6 μm sections, deparaffinized with xylene and submerged into EDTA antigenic retrieval buffer for antigenic retrieval. The tumour sections were incubated with the p21 antibody overnight at 4 °C, treated with an anti-rabbit secondary antibody for 1 h at RT and incubated with the streptavidin–horseradish peroxidase complex. The sections were stained with haematoxylin.

### In vivo analysis

Stable PANDAR-depleted AGS cells (1×10^6^) were subcutaneously injected into the right flank of 3-week-old athymic nude mice (*n* = 6). The xenografts were treated with nutlin-3 via an oral administration at 200 mg/kg once daily for 6 weeks. The nude mice were killed after 6 weeks of nutlin-3 treatment, and the tumour weight and inhibitory rate were measured and calculated.

### Optical in vivo imaging analysis

Stable PANDAR-depleted AGS cells (1×10^6^) were injected into immunodeficient mice (3 animals per group) by tail vein assays and subsequently received 200 mg/kg of an oral dose of nutlin-3 once daily. After 2 weeks, the nude mice were evaluated for lung colonization capacity. Tumour progression was monitored by imaging with a Xenogen Spectrum small animal imaging system 15 min after an injection of 100 μl Luciferin Substrate (Caliper, Hopkinton, MA) IP into the mice.

### Statistical analyses

All the data are expressed as the mean ± standard deviation. Two variables of the microarray data and qRT-PCR data were analysed using Student’s *t*-test. The categorical variables were analysed using the chi-square (*χ*^2^) test. The 5-year survival rate analyses of GC patients were performed using the log-rank (Mantel–Cox) test. The tumour weights were evaluated by Mann−Whitney t test. A *p* value of <0.05 was considered significant.

## Results

### Overexpression of PANDAR in GC patients indicates malignant transformation

**Fig. 1:**
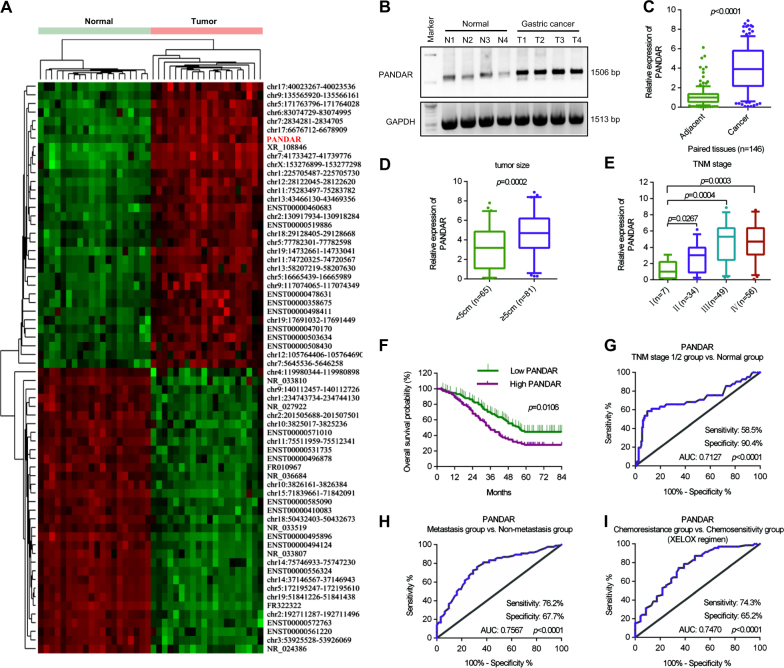
Prognostic and diagnostic significance of PANDAR for GC patients. **a** LncRNA expression profiles were evaluated by an lncRNA expression microarray. Heat map showed different lncRNA expression among samples. ‘Red’ indicates high expression; ‘Green’ indicates low expression. **b** The expression of PANDAR was demonstrated using northern blotting analysis in paired GC samples. **c** The expression of PANDAR was demonstrated using real-time PCR analysis in paired GC samples. **d** High levels of PANDAR in GC tissues correlated with increased tumour size. **e** High levels of PANDAR in GC tissues correlated with increased TNM stage. **f** Kaplan−Meier analyses of the overall survival. Patients with a high level of PANDAR expression (above mean; *n* = 72) were associated with a significantly lower overall survival rate than patients with a low level of PANDAR expression (below mean; *n* = 74). **g** ROC plots for the PANDAR discriminating the TNM stage 1/2 group from the healthy group. AUC area under the curve. **h** ROC plots for the PANDAR discriminating the metastasis group from the non-metastasis group. **i** ROC plots for the PANDAR discriminating the chemoresistance group from the chemosensitivity group. XELOX regimen: Capecitabine 825–1000 mg/m^2^ bid po d1–14 q3w; Oxaliplatin 130 mg/m^2^ ivgtt d1 q3w

We compared the lncRNA expression profiles of the GC tissues and adjacent normal tissues using unsupervised hierarchical clustering in 20 patients (Fig. 1a). We identified the top ten upregulated lncRNAs using qRT-PCR in 20 paired GC samples. As shown in Supplementary Figure 1A, the expression levels of the selected lncRNAs were identified using both study analyses. PANDAR was the most changed lncRNA among the upregulated lncRNAs. Compared with the normal samples, the upregulation of PANDAR was identified by a northern blotting analysis in the paired tumour tissues (Fig. 1b). We validated the expression level of PANDAR by a qRT-PCR analysis in 146 paired GC samples (Fig. 1c). The expression of PANDAR was closely associated with tumour size, TNM classification and N classification (Figs. 1d, e; Supplementary Table 2). Gastric cancer patients with a high expression of PANDAR showed significantly shorter 5-year overall survival rates than those with low expression of PANDAR (Fig. 1f). In the univariate and multivariable analysis, PANDAR expression, TNM classification and N classification were significant independent prognostic factors for survival time (Supplementary Table 3). Receiver operating characteristics (ROC) curves were constructed to evaluate the performance of PANDAR as a marker for discriminating the early-stage group from the healthy group (Fig. 1g**)**, the metastasis group from the non-metastasis group (Fig. 1h) and the chemoresistance group from the chemosensitive group (Fig. 1i) in GC patients. These results revealed that PANDAR mRNA levels in GC tissues might be a promising prognostic (metastasis and chemoresistance) and diagnostic (early diagnosis) indicator.

### PANDAR promotes the malignant progression of GC cells

**Fig. 2:**
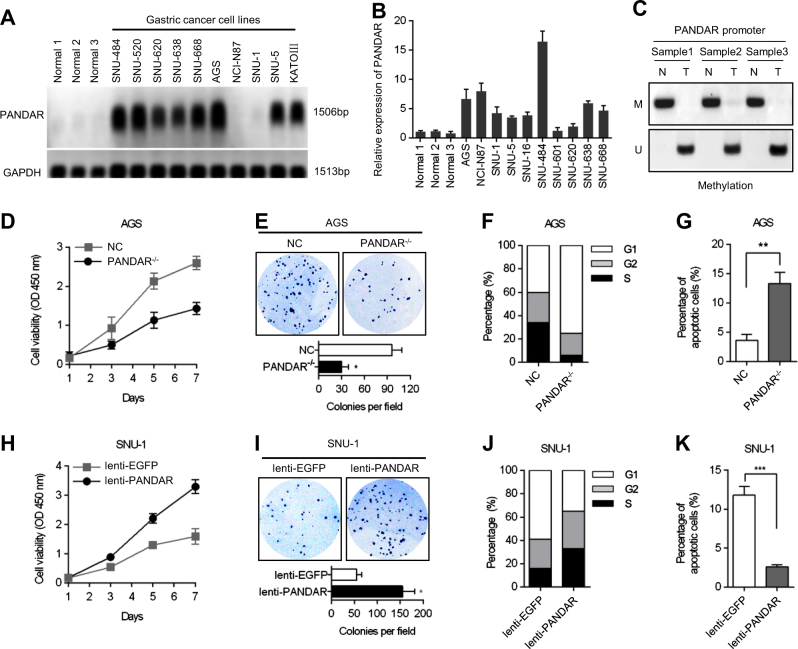
PANDAR promoted malignant progression of GC cells. **a** Northern blot analysis of PANDAR expression in normal gastric tissues and GC cell lines. **b** QRT-PCR analysis of PANDAR expression in normal gastric tissues and GC cell lines. **c** Quantitative methylation-specific PCR analysis of PANDAR promoter methylation levels in GC tissues and paired adjacent tissues. M, methylation-specific primer; U, unmethylation-specific primer. **d** Cell viability was measured by the CCK-8 assay at 1, 3, 5 and 7 days. **e** Colony formation was scored after 2 weeks. The number of colonies in the graphs was representative of three independent experiments (lower panel). **f** The effect of PANDAR knockout on the cell-cycle progression of AGS cells. **g** The effect of PANDAR knockout on sensitizing AGS cells to apoptosis. **h** Cell viability was measured by the CCK-8 assay at 1, 3, 5 and 7 days. **i** Colony formation was scored after 2 weeks. The number of colonies in the graphs was representative of three independent experiments (lower panel). **j** The effect of PANDAR overexpression on the cell-cycle progression of SNU-1 cells. **k** The effect of PANDAR overexpression on inhibiting SNU-1 cells to apoptosis. (*p < 0.05; **p < 0.01; ***p < 0.001)

We investigated the expression level of PANDAR by a northern blotting analysis and the qRT-PCR method in normal gastric tissues and in ten GC cell lines. Compared with the normal samples, the upregulation of PANDAR was identified in the majority of GC cell lines except for the NCI-N87 and SNU-1 cells (Fig. 2a, b). The demethylation of the PANDAR promoter may be responsible for PANDAR overexpression in GC tissues (Fig. 2c). Using the CRISPR/Cas9 system, the expression level of PANDAR was dramatically knocked out in AGS cells (Supplementary Figure 1B). The depletion of PANDAR obviously suppressed the proliferative and clone-forming activity of the AGS cells (Fig. 2d, e). Using flow cytometry, we found that PANDAR knockout blocked the cell-cycle progression at the G1/S checkpoint (Fig. 2f). We also identified that the depletion of PANDAR statistically promoted apoptotic activity in the AGS cells (Fig. 2g). In contrast, the exogenous expression of PANDAR, using a lentiviral expression vector (lenti-PANDAR), significantly increased the proliferation and clone-forming ability of SNU-1 cells (Supplementary Figure 1C; Fig. 2h, i). Furthermore, the overexpression of PANDAR promoted S phase entry (Fig. 2j) and suppressed the cancer cell apoptotic activity (Fig. 2k). To determine the relationship between PANDAR and the chemotherapy drug oxaliplatin, we identified the cell cytotoxicity of oxaliplatin in the cancer cells. The sensitivity of PANDAR-depleted AGS cells to oxaliplatin increased. In contrast, the sensitivity of PANDAR-overexpressed SNU-1 cells to oxaliplatin decreased. These results revealed that the knockout of PANDAR led to an increased sensitivity of GC cells to oxaliplatin (Supplementary Figure 2).

### PANDAR regulates *CDKN1A* gene transcription in a p53-dependent manner

**Fig. 3:**
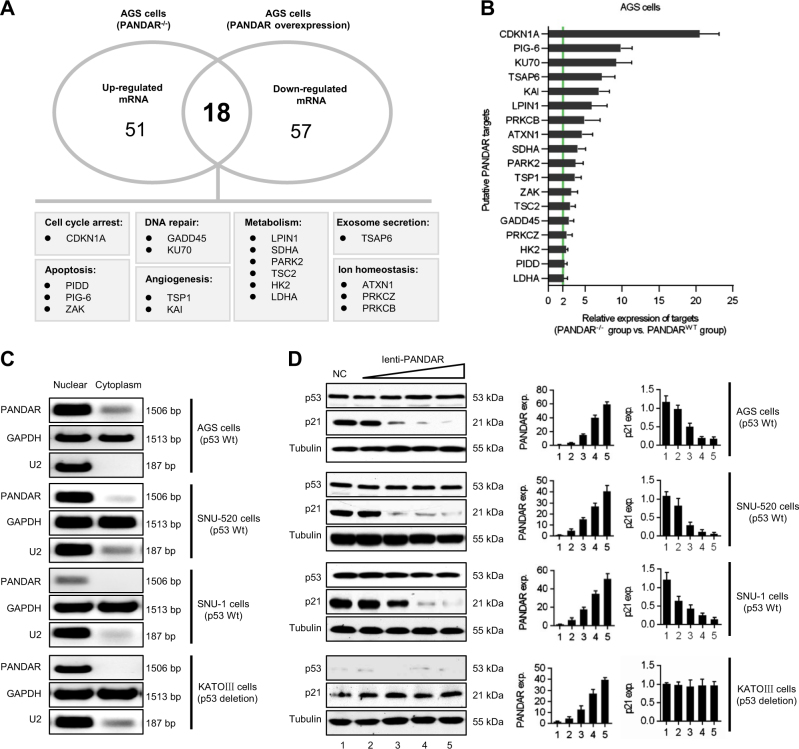
PANDAR regulated *CDKN1A* gene expression. **a** Venn diagrams show the number of differentially expressed genes from the PANDAR-depleted AGS cells and PANDAR-overexpressed AGS cells, and gene ontology showed the associated cellular pathways with these candidates. **b** QRT-PCR analysis determined the expression of putative PANDAR targets in PANDAR knockout AGS cells (PANDAR^−/−^). **c** The location of PANDAR. U2 RNA was used as a positive control for nuclear RNA. **d** Immunoblotting analysis determined the expression of p21 protein in cancer cells treated with dose-gradient increased PANDAR

To identify the functional putative targets of PANDAR, we investigated the gene expression profiles using a microarrays analysis in the PANDAR-depleted AGS cells. Compared with the negative control groups, 69 genes were upregulated and 17 genes were downregulated (Supplementary Figure 3). In contrast, we also investigated the gene expression profiles using a microarrays analysis in the AGS cells transfected with the lenti-PANDAR. Twenty-eight genes were upregulated and 75 genes were downregulated in the PANDAR overexpression groups (Supplementary Figure 4). To reduce the false-positive rate, we intersected the gene expression profiles obtained from both of the above analyses. As shown in Fig. 3a, 18 putative PANDAR targets were identified and participated in multiple biological processes. The intersection results also implied the inhibitory transcription activity for PANDAR due to only two genes being found in the opposite intersection group (Supplementary Figure 5). We then determined the expression of these candidates using qRT-PCR in the PANDAR-depleted AGS cells, and we identified that the most changed gene was *CDKN1A*, which is a key cell cycle checkpoint gene (Fig. 3b).

By isolating both nuclear and cytoplasmic RNA, we showed that PANDAR was mainly present in the nucleus (Fig. 3c). Our results showed that p21 levels were downregulated by PANDAR in a dose-dependent manner in p53 wild-type cancer cells but not in the p53 deletion mutation cancer cells (Fig. 3d). These results indicated that PANDAR regulates *CDKN1A* gene transcription in a p53-dependent manner.

### PANDAR disturbs the binding of the p53 protein with the *CDKN1A* promoter

**Fig. 4:**
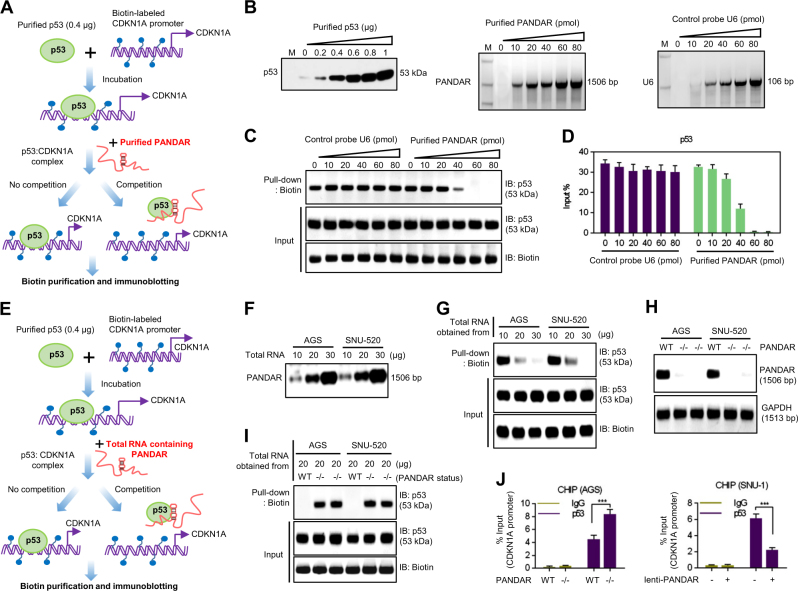
PANDAR disturbed the binding of p53 protein with *CDKN1A* promoter. **a** Schematic diagram showing the experimental design of purified PANDAR competitively binding with purified p53 protein at the *CDKN1A* promoter. We used 0.4 μg purified p53 protein and incubated it with 1 μg biotinylated *CDKN1A* promoter. After incubation, purified PANDAR or control probe U6 was added to the reaction mixture to compete with the p53:*CDKN1A* complex. Immunoblotting analysis was used to detect the residual amount of p53 protein after biotin precipitation. **b** Amount of purified p53 protein, purified PANDAR and control probe U6. M, maker. **c** Different amount of purified PANDAR and control probe U6 competing with the p53:*CDKN1A* complex. **d** The imageJ software was used to quantitate the residual amount of p53 protein after biotin precipitation. **e** Schematic diagram showing the modified experimental design of total RNA containing PANDAR competitively binding with purified p53 protein at the *CDKN1A* promoter. After incubation, different amounts of total RNA extracted from cancer cells were added to the reaction mixture to compete with the p53:*CDKN1A* complex. Immunoblotting analysis was used to detect the amount of p53 protein after biotin precipitation. **f** Amount of PANDAR in total RNA was determined by qRT-PCR. **g** Different amounts of total RNA containing PANDAR competed with the p53:*CDKN1A* complex. **h** Amount of PANDAR was determined by qRT-PCR in cancer cells treated with the CRISPR/Cas9 system for PANDAR. **i** Depletion of PANDAR in total RNA failed to compete with the p53:*CDKN1A* complex. **j** ChIP assays with an anti-p53 or negative control (anti-IgG) antibodies showed that the binding of p53 protein with the *CDKN1A* promoter was regulated by PANDAR in human GC cells. The *y*-axis represents the % input of the promoter fragments captured by the two different antibodies

We conducted a competition assay to investigate the interaction of p53 protein and PANDAR. The biotin-labelled *CDKN1A* promoter (1 μg) was synthesized and incubated with the purified p53 protein. We then added purified PANDAR produced by RNA synthesis to the reaction mixture (Fig. 4a). Before the assay, we evaluated the amount of purified p53 protein, purified PANDAR and control probe U6 used in this examination **(**Fig. 4b). After adding the variant amount of purified PANDAR or control probe U6, we found that PANDAR significantly abolished the binding of p53 protein with the *CDKN1A* promoter in the presence of 40 pmol of purified PANDAR (Fig. 4c). We also confirmed the results in quantitative assay (Fig. 4d).

Next, we modified the experimental design to determine whether the endogenic PANDAR in the cancer cells interacted with the p53 protein. The total RNA extracted from the AGS and SNU-520 cells was used instead of the purified PANDAR (Fig. 4e). We identified that 20 μg of total cellular RNA containing PANDAR successfully abrogated the binding of the p53 protein with the *CDKN1A* promoter in the AGS and SNU-520 cells (Fig. 4f, g). After PANDAR knockout (Fig. 4h), the p53 protein maintained its interaction with the *CDKN1A* promoter to form the p53:*CDKN1A* complex (Fig. 4i). We also performed chromatin immunoprecipitation experiments to determine the influence of PANDAR on the p53:*CDKN1A* promoter complex. We defined the upstream 3000 bases of the first exon of the *CDKN1A* gene as the *CDKN1A* promoter. As shown in Fig. 4j, the depletion of PANDAR increased the binding of the p53 protein to the *CDKN1A* promoter. However, the overexpression of PANDAR decreased the binding of the p53 protein to the *CDKN1A* promoter.

### PANDAR directly interacts with the p53 protein

**Fig. 5:**
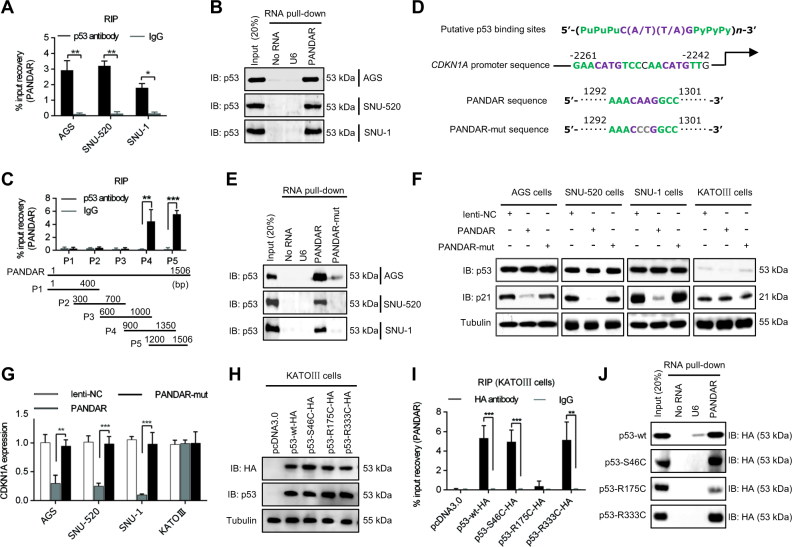
PANDAR directly interacted with p53 protein. **a** RIP analysis determined the recovery of PANDAR in cancer cells using p53 antibody. **b** RNA pull-down analysis determined the p53 protein−PANDAR interaction using 5′ biotin-linked RNAs. U6 RNA as a negative control. **c** RIP analysis determined the recovery of a portion of PANDAR in AGS cells using p53 antibody. **d** Alignment and conserved putative p53 binding sites in *CDKN1A* promoter and PANDAR nucleic acid sequence. **e** RNA pull-down analysis determined the p53 protein−PANDAR interaction using 5’ biotin-linked RNAs. **f** Immunoblotting analysis determined the levels of p21 protein in cancer cells treated with different expression vectors. **g** QRT-PCR analysis determined the expression of *CDKN1A* mRNA in cancer cells treated with different expression vectors. **h** Immunoblotting analysis determined the p53 protein levels using the p53 antibody and HA antibody. **i** RIP analysis determined the recovery of PANDAR in KATOIII cells transfected with different mutated-*P53* gene expression vectors using HA antibody. **j** RNA pull-down analysis determined the PANDAR-p53 protein interaction using protein lysis of KATOIII cells transfected with different mutated-*TP53* gene expression vectors

To investigate whether the biological effect of PANDAR was related to its binding ability with p53 protein, we performed RNA immunoprecipitation (RIP) experiments with p53 wild-type cancer cells (AGS, SNU-520 and SNU-1) (Fig. 5a). Our results clearly showed that PANDAR was obviously recovered with the p53 protein. In contrast, we performed an RNA pull-down assay using various species of 5′ biotin-linked RNAs. As shown in Fig. 5b, the p53 protein was only pulled down by PANDAR but not by the negative control (U6). To investigate the region of PANDAR responsible for the binding activity of the p53 protein, we designed various primers to detect the different fragments of PANDAR. Both part 4 (P4) and part 5 (P5) of PANDAR were markedly recovered by the p53 protein (Fig. 5c). In further analyses, we identified a nucleotide sequence which might be responsible for binding with the p53 protein in PANDAR (Fig. 5d). After mutation at this p53 putative binding site, the p53 protein could not be effectively pulled down by PANDAR (Fig. 5e). The PANDAR mutation also abrogated its competitive inhibitory ability towards the p53 protein in the p53 wild-type cancer cells (AGS, SNU-520 and SNU-1) (Fig. 5f). We also showed that the overexpression of PANDAR reduced the expression of *CDKN1A*, whereas the overexpression of mutant PANDAR did not affect the *CDKN1A* expression at the mRNA level (Fig. 5g). These results indicated that PANDAR interacted with the p53 protein through its 1292–1301 bp nucleic acid sequence (aaaCAAGgcc).

To investigate which motif of p53 protein was responsible for the binding activity towards PANDAR, we constructed a series of *TP53*-mutated expression vectors. We first determined the protein expression levels of these *TP53*-mutated expression vectors in KATOIII cells (Fig. 5h). Then, we performed an RIP analysis with the KATOIII cells (p53 deletion) transfected with different *TP53*-mutated expression vectors using the HA-antibody (Fig. 5i). Our results showed that the DNA-binding domain mutation (R175C) suppressed the recovery of PANDAR with the p53 protein. Additionally, using 5′ biotin-linked RNAs, we performed an RNA pull-down analysis to define the binding activity of the mutated-p53 protein with PANDAR. The mutation at the DNA-binding domain of the p53 protein abrogated its ability to interact with PANDAR (Fig. 5j). These results illustrated that PANDAR inhibited *CDKN1A* gene transcription by competitively binding with the p53 protein.

### PANDAR depletion, combined with nutlin-3, inhibits proliferation and induces apoptosis in GC cells in vivo

To determine whether PANDAR regulated the transcription of multiple targets mainly by interacting with the p53 protein, we performed a microarray analysis using the AGS cells in which p53 was overexpressed (Supplementary Figure 6). As shown in Supplementary Figure 7A, a large body of the PANDAR putative targets (53/69, 76.8%) overlapped with the p53 targets. Furthermore, we confirmed the regulation of randomly selected p53 targets by PANDAR knockout (Supplementary Figure 7B). We also performed a linear regression analysis for PANDAR and the randomly selected p53 targets in GC tissues (Supplementary Figure 7C). These results illustrated that PANDAR controlled the transcription of multiple targets mainly by interacting with p53.

To further identify the roles of *CDKN1A* and *TP53* in tumour suppression, we first knocked out the *CDKN1A* gene or *TP53* gene expression by the CRISPR/Cas9 system. The depletion of the *CDKN1A* gene and *TP53* gene dramatically promoted cancer cell growth (Supplementary Figure 8A). In addition, the upregulation of PANDAR lost their ability to promote tumour growth in the *TP53*-depleted AGS cells (Supplementary Figure 8B). These results indicated that PANDAR promoted tumourigenesis in a p53-dependent manner.

**Fig. 6:**
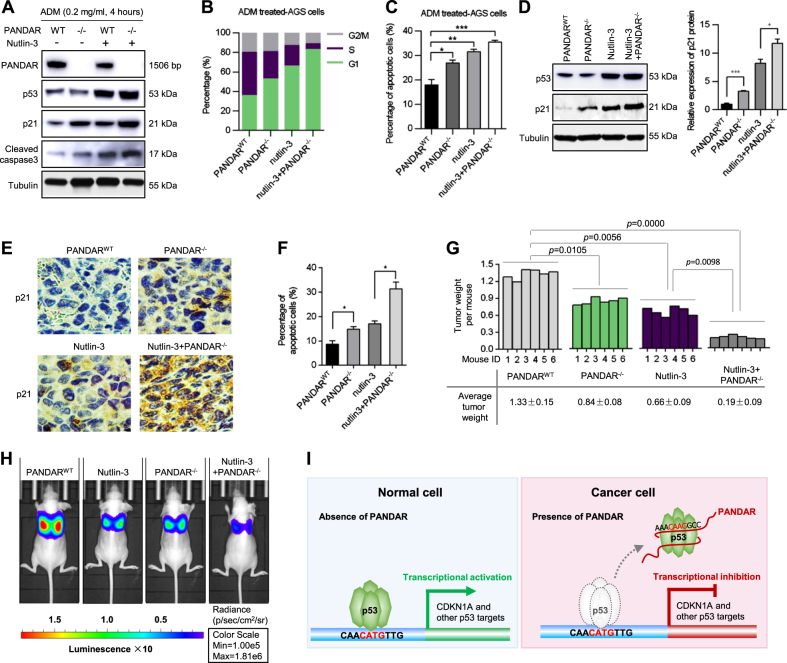
PANDAR depletion combined with nutlin-3 synergistically inhibited proliferation and induced apoptosis in GC cells. **a** After treated with nutlin-3 (10 μm) and ADM (0.2 mg/ml) for 4 h, PANDAR-depleted AGS cells were analysed by immunoblot analysis. WT, wild type; −/−, knockout. **b** After treated with nutlin-3 and ADM for 4 h, PANDAR-depleted AGS cells were analysed by flow cytometry analysis. **c** The effect of PANDAR on affecting AGS cells to ADM-induced apoptosis. **d** Immunoblotting analysis determined the p53 and p21 protein levels of AGS cells treated with CRISPR/Cas9 system for PANDAR and nutlin-3 (10 µm) for 72 h. **e** Xenografts treated with nutlin-3 via oral administration at 200 mg/kg once daily for 6 weeks. IHC analysis determined the p21 protein levels in tumour tissues. **f** The presence of apoptotic cells in the representative tumour tissues was detected by a TUNEL assay. **g** Anti-tumour effect of PANDAR depleting combined with nutlin-3. The bar graph shows the average tumour weight per mouse and the standard deviation. **h** Stable PANDAR-depleted AGS cells were injected into immunodeficient mice (three animals per group) by tail vein assays and subsequently received 200 mg/kg of an oral dose of nutlin-3 once daily. After 2 weeks, nude mice were evaluated for lung colonization capacity. **i** A model illustrating the putative roles of PANDAR in controlling the transcription of *CDKN1A* gene through competitive binding with p53 protein

To observe whether the combined treatment of PANDAR depletion with the p53 activator nutlin-3 caused any changes in the stress response of the cells, we first used the CRISPR/Cas9 system to knock out PANDAR expression in the AGS cells (Supplementary Figure 8C). Then, the PANDAR-depleted AGS cells were treated with nutlin-3 and adriamycin (ADM) for 4 h. The knockout of PANDAR combined with nutlin-3 dramatically increased p21 and cleaved caspase3 protein expression in the ADM-treated AGS cells (Fig. 6a). Using flow cytometry, we found that the combination of PANDAR depletion and nutlin-3 blocked the cell-cycle progression at the G1/S checkpoint (Fig. 6b) and increased the apoptotic activity of the AGS cells (Fig. 6c).

We investigated whether PANDAR depletion combined with the p53 protein activator had a synergistic effect on cancer therapy. PANDAR depletion combined with nutlin-3 obviously promoted an increase in the p53 protein level and p21 protein level in the AGS cells (Fig. 6d). After a subcutaneous injection of the PANDAR-depleted AGS cells and treatment with nutlin-3 for 6 weeks, the PANDAR depletion combined with nutlin-3 group showed increased levels of the p21 protein (Fig. 6e). The combination of PANDAR depletion and nutlin-3 increased AGS cell apoptosis (Fig. 6f). Additionally, the knockout of PANDAR combined with nutlin-3 reduced the tumour weight of the mice compared with the negative control-treated group (Fig. 6g). The PANDAR-depleted AGS cells were injected into the tail vein of immunodeficient mice. The cell survival in the circulation and extravasation to and growth in the lungs were evaluated and analysed. After 2 weeks, we observed that the knockout of PANDAR combined with nutlin-3 remarkably inhibited the lung colonization capacity (Fig. 6h). These data indicated that a combination of PANDAR depletion and nutlin3 treatment has therapeutic potential in GC cells.

## Discussion

PANDAR is a relatively novel lncRNA that plays an important role in the development of multiple cancers. Previous studies have reported that PANDAR was significantly upregulated in cholangiocarcinoma (CCA) patients^21^, bladder cancer patients^22^, clear cell renal cell carcinoma (ccRCC) patients^23^, hepatocellular carcinoma^24^ and colorectal cancer patients^25^. The overexpression of PANDAR in cancer patients was closely associated with a higher histological grade, advanced TNM stage and poorer overall survival. High expression of PANDAR in GC patients was correlated with the depth of invasion, advanced TNM stage and lymphatic metastasis. Importantly, a high expression of PANDAR could serve as an independent unfavourable prognostic role in GC^26^.

In this study, we first investigated the different lncRNA expression patterns between GC tissues and paired adjacent normal tissues. A clinical pathology characteristic correlation analysis revealed that the upregulated PANDAR was associated with a statistically significant increased tumour size, TNM classification, N classification and a poor survival rate in GC patients. ROC curves identified that the PANDAR levels were a marker for discriminating the early-stage tumour group from the healthy group, the metastasis group from the non-metastasis group and the chemoresistance group from the chemosensitive group in GC patients. Our results suggested that PANDAR is a new diagnostic and therapeutic marker for patients with GC.

Quite a few lncRNAs affect gene transcription via chromatin modification^27,28^, transcription machinery modulation^29^, or specific transcription factor modulation^30^, which implies that the dysregulated lncRNAs may have regulatory roles in cancer progression. PANDAR is induced in response to DNA damage and represses apoptosis by inhibiting the function of the transcription factor nuclear transcription factor Y subunit alpha (NF-YA). Further experiments demonstrated that PANDAR expression was induced by p53 protein, and chromatin immunoprecipitation assays confirmed that PANDAR was a direct transcriptional target of p53 protein. In turn, the stability of the p53 protein was markedly reduced by PANDAR silencing. PANDAR stabilizes the p53 protein in response to DNA damage and provides new insight into the regulatory mechanisms of p53^31,32^. PANDAR promotes the G1-S transition by suppressing cyclin-dependent kinase inhibitor p18 or p16 (INK4A) expression^33,34^. In our study, we determined that PANDAR inhibited *CDKN1A* gene transcription via a competitive interaction with the p53 protein. Mechanically, we identified that PANDAR combined with the p53 protein through its 3′-terminal sequence of nucleic acids. In turn, the p53 protein interacted with PANDAR via its DNA binding motif. PANDAR inhibited *CDKN1A* gene transcription by competitively binding to the p53 protein, acting as a p53-response element decoy. In addition, PANDAR controlled its multiple targets mainly by interacting with the p53 protein.

*TP53* functions as a tumour suppressor, and almost half of all human cancers harbour somatic mutations in this gene^35^. There is an urgent need to develop p53-targeting therapy as an approach to treat human cancers. However, p53-targeting therapy alone does not exhibit exciting efficacies in cancer treatment due to the limitation of the adenovirus delivery^36^, the lack of an apoptotic response^37^ and the acquired resistance to p53-targeted therapy^38^. A new opportunity to be investigated is the combination of p53-targeting therapy with conventional chemotherapeutic drugs or other molecularly targeted therapies. Therefore, whether PANDAR depleting combined with a p53 activator might be a benefit for GC therapy was worth investigating. The protein level of p21 in the nude mouse tumour samples was significantly increased in the PANDAR-depletion, nutlin3-treated group, and this combined therapeutic method obviously reduced the nude mouse tumour size and tumour weight in vivo. Additionally, the knockout of PANDAR, combined with nutlin-3, remarkably inhibited the AGS cell lung colonization capacity. These results implied that the therapeutic efficacy of drugs for p53 activation may be strengthened by the RNA interference of lncRNAs.

A significant proportion of studies provide a role for p53 in the regulation of cell cycle and cell proliferation. Therefore, the identification of lncRNAs regulating p53 function shows a promising potential for the molecular targeting of GC via restoration of p53 activity. Previous studies confirm that the lncRNAs HOTAIR and PANDAR are direct transcriptional targets of p53. Inversely, upregulated HOTAIR inhibits the expression of p53 by enhancing the p53 promoter histone H3 lysine 27 trimethylation (H3K27me3), and the overexpression of PANDAR increases the stability of the p53 protein in response to DNA damage^31,39^. Here, we present a composite study involving the identification of PANDAR and elucidating its tumour promoting response by competitively binding with p53. PANDAR is a bidirectional transcript from the CDKN1A promoter. The expression of PANDAR and CDKN1A are positively regulated by p53^40^. Since CDKN1A mediates cell cycle arrest, PANDAR is able to block apoptosis through its interaction with p53 to limit the expression of CDKN1A and other pro-apoptotic genes. Similar to PANDAR, lincRNA-p21 also serves as a repressor in p53-dependent transcriptional responses by directing the recruitment of hnRNP K to its genomic targets^41^. A number of other lncRNAs are also under direct p53 regulation or show correlations with p53 levels. The lncRNA MALAT1 is required for G1/S and mitotic progression, and p53 is a major downstream mediator of MALAT1 activity^12^. As a tumour suppressor, MEG3 inhibits tumour cell proliferation possibly through the induction of apoptosis. MEG3 stimulates p53-mediated transactivation in human meningioma cell lines^42^. Here, we discuss how lncRNAs serve as p53 regulators or p53 effectors. Further characterization of these p53-associated lncRNAs in cancer will provide a better understanding of lncRNA-mediated gene regulation in the p53 pathway.

In this study, upregulated PANDAR was identified in the GC samples. PANDAR bound to the DNA-binding domain of the p53 protein by acting as a p53-response elements decoy, thus competing with the *CDKN1A* promoter for binding to the p53 protein. Through this unique transcriptional modification pattern, PANDAR notably facilitated cancer cell proliferation, clone formation and chemotherapy resistance. Importantly, PANDAR depletion combined with a p53 activator showed perfect efficacy in cancer treatment in vivo. Our results imply that PANDAR is not only a powerful diagnostic biomarker for GC patients but also a promising potential therapeutic target for cancer treatment.

## Electronic supplementary material

Supplementary file

Supplementary figures
