# Supplementary material for: Long noncoding RNA PANDAR blocks CDKN1A gene transcription by competitive interaction with p53 protein in gastric cancer
Source: Cell Death Dis. 2018 Feb 7;9(2):168. doi: 10.1038/s41419-017-0246-6 (PMC5833854; doi:10.1038/s41419-017-0246-6)
Supplement: Supplementary file 1 — Supplementary file [file 41419_2017_246_MOESM1_ESM.docx]

**Long Noncoding RNA PANDAR Blocks *CDKN1A* Gene Transcription by Competitive Interaction with p53 Protein in Gastric Cancer**

Jun Liu^1#^, Qiwen Ben^3#^, Xiangyi He^3^, Xiaoqun Yang^4^, Jun Ma^5^, Wen Zhang^5^, Zhiming Wang^6^, Tianshu Liu^6^, Jianjun Zhang^2*^, Hongxia Wang^1*^

Supplementary figure 1 is related to figure 1;

Supplementary figure 2 is related to figure 2;

Supplementary figure 3 is related to figure 3;

Supplementary figure 4 is related to figure 3;

Supplementary figure 5 is related to figure 3;

Supplementary figure 6 is related to figure 6;

Supplementary figure 7 is related to figure 6;

Supplementary figure 8 is related to figure 6;

Supplementary Table 1 is related to figure 1;

Supplementary Table 2 is related to figure 1;

Supplementary Table 3 is related to figure 1;

Supplementary Table 4 lists all primers used in this study.

**Materials and Methods**

**Microarray analysis**

Gene expression profiles were investigated using the Agilent Human gene array 8*60 K (OE Biotech, Shanghai, China). The RNA samples were first reverse transcribed into cDNA, and these cDNA samples were then labeled using a Low Input Quick-Amp Labeling Kit (Agilent Technologies, Santa Clara, CA, USA). Labeled cDNA samples were used as probes to hybridize to lncRNA microarrays. After the samples were hybridized, the microarrays were scanned with an Agilent microarray scanner. Feature Extraction software (version 10.7.1.1, Agilent Technologies) was used to analyze array images to obtain raw data. Gene-Spring software (version 12.5, Agilent Technologies) was employed to finish the basic analysis of the raw data. Initially, the raw data were normalized using the quantile algorithm. The probes with at least 100% of samples in any one condition out of two conditions having flags that indicate ‘Detected’ were chosen for further data analysis. Differentially expressed genes were then identified through fold change, and P values were calculated using t-tests. The thresholds set for up and down-regulated genes were a fold change ≥2.0 and a P value ≤0.05.

**Cell cytotoxicity assay**

AGS cells and SNU-1 cells (1×10^3^ cells/well) were incubated with 100μl culture medium in 96-well plates at 37℃ in a CO2 incubator for 24 h. Escalated doses of oxaliplatin **(0.19111, 0.3828125, 0.765625, 1.53125, 3.0625, 6.125, 12.5, 25, 50 and 100 μM**, respectively**) were added to each well and incubated with the tumor cells for different times (24, 48, 72, 96 and 120 h**, respectively**).** Cell growth inhibition was assessed by using Cell Counting Kit-8 (Dojindo, Japan) as following: CCK-8 (10 **μ**L) was added into each well. After 1 h incubation at 37℃, absorbance at 450 nm wavelength was measured. Background absorbance from empty wells was subtracted from sample wells. Cell growth activity was expressed as the absorbance at 450 nm, and the concentration of oxaliplatin resulting in 50% growth inhibition (IC50) was calculated. All experiments were performed in triplicate.

**Statistical analyses**

All statistical analyses were carried out using SPSS software (version 18 for Windows; SPSS, Chicago, IL). The association of PANDAR expression with various clinicopathologic features was analyzed using the chi-square (χ^2^) test. A Cox proportional hazard model (backward, stepwise) for multivariable analysis was applied for factors that achieved significance in univariable analysis. A *p*-value of <0.05 was considered significant.

**Figure legends:**

**Supplementary Figure 1.** Validation of lncRNAs expression in GC tissues and GC cell lines**.**

1. Top ten up-regulated expression of lncRNAs was demonstrated using qRT-PCR analysis in GC samples (n=146).
2. **QRT-PCR analysis of** PANDAR **expression in AGS cells treated with CRISPR/Cas9 system.**
3. **QRT-PCR analysis of** PANDAR **expression in SNU-1 cells treated with lenti-PANDAR.**

**Supplementary Figure 2.** **Sensitivity of gastric cancer cells to oxaliplatin was related with PANDAR expression.**

1. **Cell growth inhibition was evaluated by the Cell Counting Kit-8 (Dojindo, Japan). After depletion of PANDAR, AGS cells were treated with escalated doses of oxaliplatin (0.19111, 0.3828125, 0.765625, 1.53125, 3.0625, 6.125, 12.5, 25, 50 and 100 μM) and incubated for (24, 48, 72, 96 and 120 h). IC50 was calculated, and all experiments were performed in triplicate.**
2. **After transfected with lenti-PANDAR vector, SNU-1 cells were treated with escalated doses of oxaliplatin (0.19111, 0.3828125, 0.765625, 1.53125, 3.0625, 6.125, 12.5, 25, 50 and 100 μM) and incubated for (24, 48, 72, 96 and 120 h). IC50 was calculated, and all experiments were performed in triplicate.**

**Supplementary Figure 3. The gene expression profiles of AGS cells that PANDAR was knocked out.**

**Supplementary Figure 4. The gene expression profiles of AGS cells transfected with PANDAR expression vector (lenti-PANDAR).**

**Supplementary Figure 5. Venn diagrams show the number of differentially expressed genes from the PANDAR-depleted AGS cells and PANDAR-overexpressed AGS cells.**

**Supplementary Figure 6. The gene expression profiles of AGS cells treated with p53 expression vector.**

**Supplementary Figure 7.** PANDAR regulated multiple targets expression through interacting with p53 protein.

1. Venn diagrams show the number of differentially expressed genes from the PANDAR-depleted AGS cells and p53-overexpressed AGS cells.
2. Immunoblotting analysis determined the regulation of p53 targets by PANDAR in p53 knockout AGS cells.
3. Linear regression analysis determined the correlation of PANDAR with p53 targets in gastric cancer tissues (n=146).

**Supplementary Figure 8. CCK-8 analysis performed in AGS cells.**

1. Depletion of *CDKN1A* gene and *TP53* gene dramatically promoted cancer cells growth.
2. Up-regulation of PANDAR lost their ability to promote tumor growth in *TP53* gene knockout AGS cells.
3. QRT-PCR analysis determined the PANDAR expression after treatment of PANDAR-depleted AGS cells with nutlin-3 (10 µM) for 72 hours.

**Supplementary Table 1: The overall survival data of GC patients.**

| **TNM stage** | **Treatment** | **High PANDAR levels (above mean)** | | **Low PANDAR levels (below mean)** | |
| --- | --- | --- | --- | --- | --- |
|  |  | **No. patients** | **Overall survival (months)**  **(mean±SD)** | **No. patients** | **Overall survival (months)**  **(mean±SD)** |
| Ⅰ | D2 lymphadenectomy  + postoperative follow-up | 0 | / | 7 | 68.9**±**9.2 |
| Ⅱ | D2 lymphadenectomy +  chemotherapy regimen: XELOX**^#^** | 8 | 46.6**±17.2** | 26 | 58.0**±**21.1 |
| Ⅲ |  | 30 | 42.8**±23.2** | 19 | 47.2**±**22.9 |
| Ⅳ | chemotherapy regimen: XELOX^#^ | 34 | 30.0**±16.6** | 22 | 35.4**±**15.3 |
| Total | | 72 |  | 74 |  |

^#^ XELOX regimen: **Capecitabine**825-1000mg/m^2^ bid po d1-14 q3w; **Oxaliplatin** 130mg/m^2^ ivgtt d1 q3w.

**Supplementary Table 2. Clinicopathological correlation of PANDAR expression in gastric cancer.**

| **Clinicopathological features** | **No. of cases** | **%** | **PANDAR** | | ***P* value** |
| --- | --- | --- | --- | --- | --- |
|  |  |  | **Up-regulation**  **(above mean)** | **Down-regulation**  **(below mean)** |  |
| Age (years) |  |  |  |  | 0.6877 |
| ≥60 | 75 | 51.37 | 40 | 35 |  |
| <60 | 71 | 48.63 | 32 | 39 |  |
| Gender |  |  |  |  | 0.0581 |
| Male | 93 | 63.70 | 40 | 53 |  |
| Female | 53 | 36.30 | 32 | 21 |  |
| Tumor size |  |  |  |  | **< 0.0001** |
| ≥5 cm | 81 | 55.48 | 57 | 24 |  |
| <5 cm | 65 | 44.52 | 15 | 50 |  |
| Site |  |  |  |  | 0.8484 |
| Cardia-fundus | 81 | 45.89 | 40 | 41 |  |
| Corpus | 19 | 19.18 | 9 | 10 |  |
| Antrum | 40 | 23.97 | 19 | 21 |  |
| two or more sites | 6 | 10.96 | 4 | 2 |  |
| TNM stage |  |  |  |  | **< 0.0001** |
| T1 | 7 | 4.79 | 0 | 7 |  |
| T2 | 34 | 23.28 | 8 | 26 |  |
| T3 | 49 | 33.56 | 30 | 19 |  |
| T4 | 56 | 38.36 | 34 | 22 |  |
| Pathologic grade |  |  |  |  | **< 0.0001** |
| N1 | 45 | 30.82 | 10 | 35 |  |
| N2 | 59 | 40.41 | 28 | 31 |  |
| N3a | 29 | 19.86 | 23 | 6 |  |
| N3b | 13 | 8.9 | 11 | 2 |  |
| Intravascular embolus |  |  |  |  | 0.4627 |
| No | 121 | 82.88 | 58 | 63 |  |
| Yes | 25 | 17.12 | 14 | 11 |  |

**Supplementary Table 3. Univariate and multivariate analysis of different prognostic variables in patients with gastric cancer by cox regression analysis.**

|  | variable | **Univariate analysis** | | | **Multivariate analysis** | | |
| --- | --- | --- | --- | --- | --- | --- | --- |
|  |  | Hazard ratio | 95% confidence  interval | P-value | Hazard ratio | 95% confidence  interval | P-value |
| Age | <60 yr vs. ≥60 yr | 0.721 | 0.368-1.995 | 0.3926 |  |  |  |
| Gender | Male vs. female | 0.974 | 0.734-1.872 | 0.1658 |  |  |  |
| Tumor size | <5 cm vs. ≥5 cm | 3.782 | 3.158-4.310 | **0.0482** | 2.626 | 2.076-3.256 | 0.0615 |
| TNM stage | T1&2 vs. T3/T4 | 4.117 | 3.531-4.639 | **0.0049** | 4.351 | 3.788-5.065 | **0.0113** |
| Pathologic grade | N1&2 vs. N3a/3b | 3.674 | 3.104-4.185 | **0.0231** | 3.208 | 2.563-4.120 | **0.0158** |
| Intravascular embolus | No vs.  Yes | 0.889 | 0.563-1.359 | 0.7340 |  |  |  |
| **PANDAR expression** | Low vs. High | 3.350 | 3.043-3.998 | **0.0146** | 3.096 | 2.697-3.536 | **0.0307** |

**Supplementary Table 4: Primers used in this study.**

| **Gene** |  | **Primer Sequence (5'->3')** | **Note** |
| --- | --- | --- | --- |
| XR_108846 | forward | TGGATCACACTCATGGGAAA | For qRT-PCR analysis (Supplementary Figure 1B) |
|  | reverse | CACCACTTGTGGCTTTTGAA |  |
| chr7:41733427-41739776 | forward | CCTTTCCAGTCAGGTGGGTC |  |
|  | reverse | CCCAGGGGAAGCATCACTTT |  |
| chr7:2834281-2834705 | forward | CTAGCACAATGCCGTGGAGA |  |
|  | reverse | GTGGGTGGTCTCTTGTTGCC |  |
| chrX:153276899-153277298 | forward | GAAGGCCTACAAGCAAAGCG |  |
|  | reverse | GGATGGCTCTCCTGATTGGG |  |
| chr17:6676712-6678909 | forward | CAGAGTCCTCAGCAGCCAAA |  |
|  | reverse | TGCTGGGTTCCACAAACAGT |  |
| chr5:171763796-171764028 | forward | AGCTTGAGTACTGATGGCCC |  |
|  | reverse | TCCCCCTATCCTGCTTGGAA |  |
| chr12:28122045-28122620 | forward | CAGACTAGCCTCGAGTTACACA |  |
|  | reverse | TCTTTCAGAAGCCCTTTTGTTGA |  |
| chr19:14732661-14733041 | forward | AGACTCTATGGGGATATGGGCT |  |
|  | reverse | AGAACACACAGCTGGAGGC |  |
| chr17:40023267-40023536 | forward | GGAAACTTGATCTGCGTGGC |  |
|  | reverse | GTCCAAGGGAAAGTCTGGCA |  |
| PANDAR | forward | TCCCAACAAACAAGGGGTGG |  |
|  | reverse | GTGGCCAAAGGATCTGACGA |  |
| CDKN1A | forward | AGTCAGTTCCTTGTGGAGCC | For qRT-PCR analysis (Fig.3B) |
|  | reverse | CATTAGCGCATCACAGTCGC |  |
| PIG-6 | forward | GATTGTGGTGTGCGAGAGGT |  |
|  | reverse | GTTCATTGGGATCTCCGTGC |  |
| KU70 | forward | GAAGCAAAAGGCCCAAGGTG |  |
|  | reverse | AGCAGCTCCTGCTTCTTCAG |  |
| TSAP6 | forward | CTTCGCCGCGGACCTTC |  |
|  | reverse | GGCTACTATCGCTGTCCACC |  |
| KA1 | forward | GAGCACAGAGGAGGGAATCG |  |
|  | reverse | CTGTTCCTCTCCCGCATACC |  |
| LPIN1 | forward | GTTTGCAATACAAAGGCGGC |  |
|  | reverse | GAGCTCCTTCACGGTGACAA |  |
| PRCKB | forward | GACCAAACACCCAGGCAAAC |  |
|  | reverse | GATGGCGGGTGAAAAATCGG |  |
| ATXN1 | forward | CTCGCCAGGAGGCATAATGT |  |
|  | reverse | AGGGATGTCGTCCAAGAGGA |  |
| SDHA | forward | GCTGTGGCCCTGAGAAAGAT |  |
|  | reverse | TCTCTGAAATGCCAGGCAGG |  |
| PARK2 | forward | ATGAATGCAACTGGAGGCGA |  |
|  | reverse | GACTTCCAGCTGGTGGTGAG |  |
| TSP1 | forward | CAGGAGCAACCTCTACTCCG |  |
|  | reverse | CAGCAGGGATCCTGTGTGTA |  |
| ZAK | forward | TACACAACAAGGCGGAGTGG |  |
|  | reverse | TTGCAGCAAGAGGCAAGAGA |  |
| TSC2 | forward | CGAGGCCTGCCCAACAAG |  |
|  | reverse | CTCTGGCTCCATGTAGTCGC |  |
| GADD45 | forward | CACTGTCGGGGTGTACGAAG |  |
|  | reverse | GTTGATGTCGTTCTCGCAGC |  |
| PRKCZ | forward | ATCAAGTCCCACGCGTTCTT |  |
|  | reverse | ATCCTCATCGTCTGGGGTCA |  |
| HK2 | forward | TCCATCTGCCTGTCCATGTC |  |
|  | reverse | ACTCCAGTATTGCAGGTTCCA |  |
| PIDD | forward | CAGGACCCGGAATGACAACA |  |
|  | reverse | CTTTGACCCCAGGATGACCC |  |
| LDHA | forward | ACGTGCATTCCCGATTCCTT |  |
|  | reverse | AACAGCACCAACCCCAACAA |  |
| CDKN1A promoter | forward | AGACCTGGAGCTCTCTTCCG | For ChIP analysis (Fig.4J) |
|  | reverse | CAAACAAGGGGTGGTTTGTCTG |  |
| CDKN1A | Guide sequence | GATGTCCGTCAGAACCCATG NGG | For Crispr/Cas9 system |
| TP53 | Guide sequence | GCTTGTAGATGGCCATGGCG NGG |  |
| PANDAR | Guide sequence | CGTGCACACATTTAACCCGA AGG |  |
