# Supplementary material for: Long noncoding RNA PANDAR blocks CDKN1A gene transcription by competitive interaction with p53 protein in gastric cancer
Source: Cell Death Dis. 2018 Feb 7;9(2):168. doi: 10.1038/s41419-017-0246-6 (PMC5833854; doi:10.1038/s41419-017-0246-6)
Supplement: Supplementary file 2 — Supplementary figures [file 41419_2017_246_MOESM2_ESM.pdf]

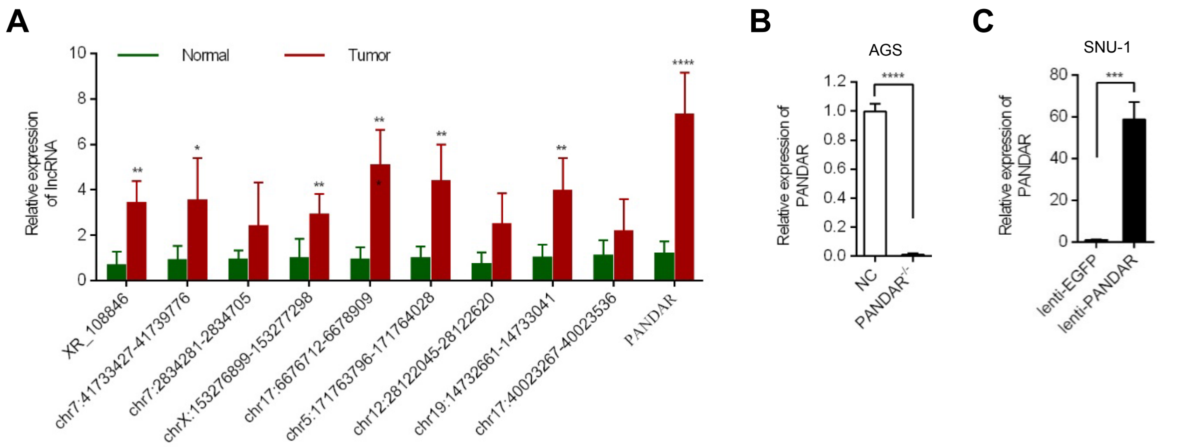

Supplementary Figure 1

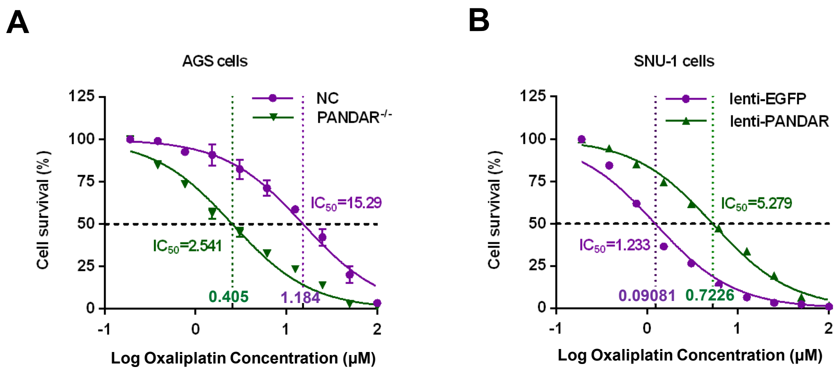

Supplementary Figure 2

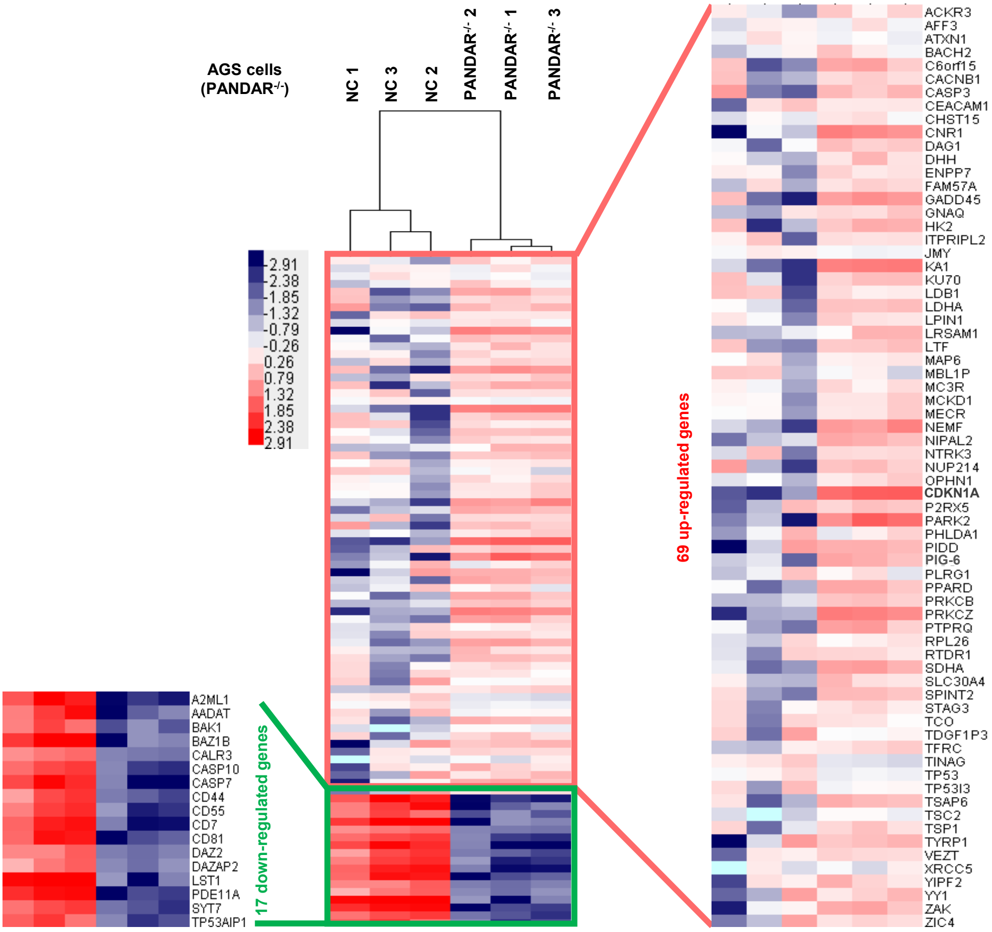

Supplementary Figure 3

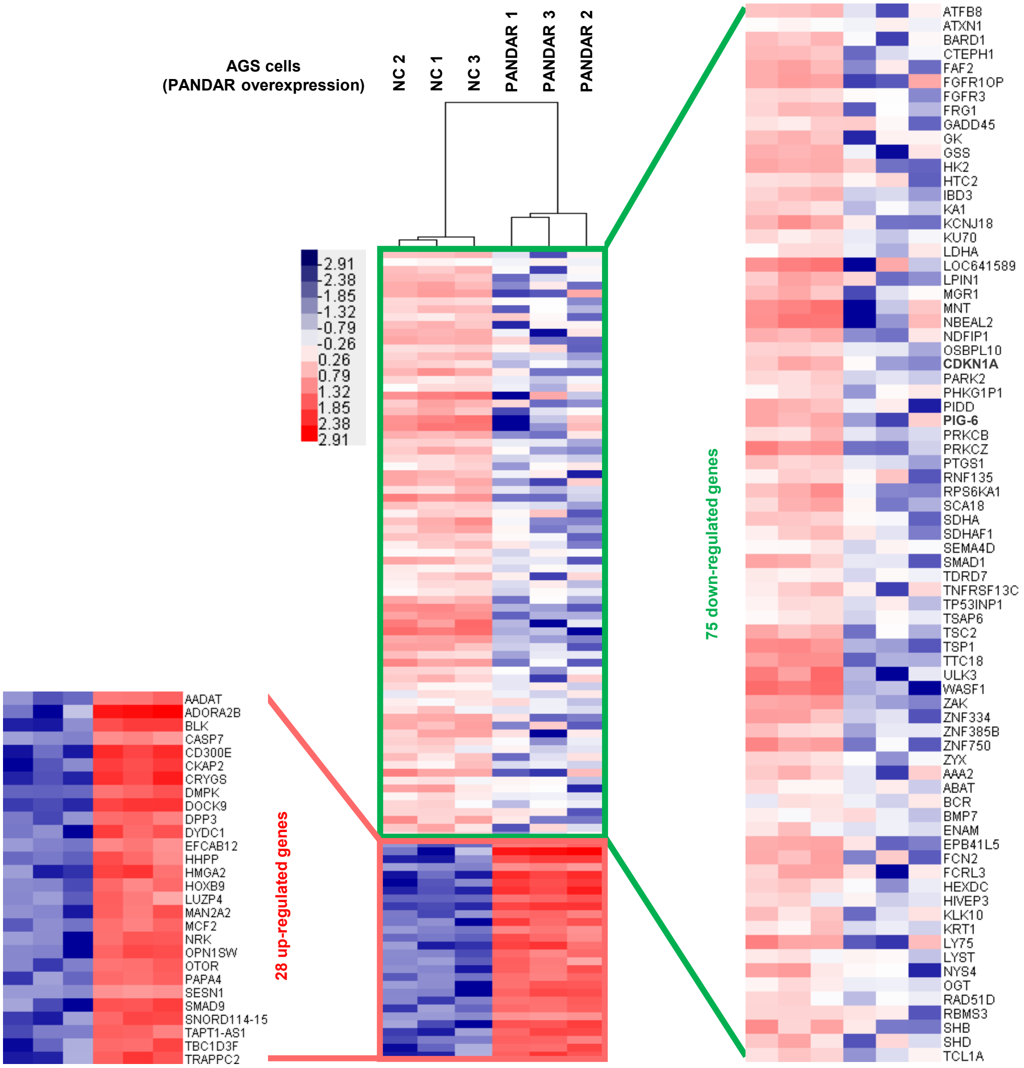

Supplementary Figure 4

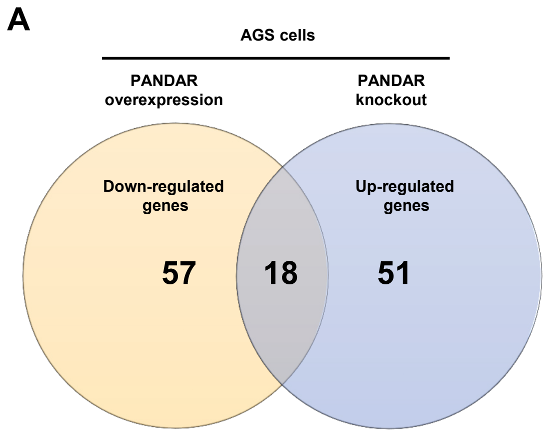

Supplementary Figure 5

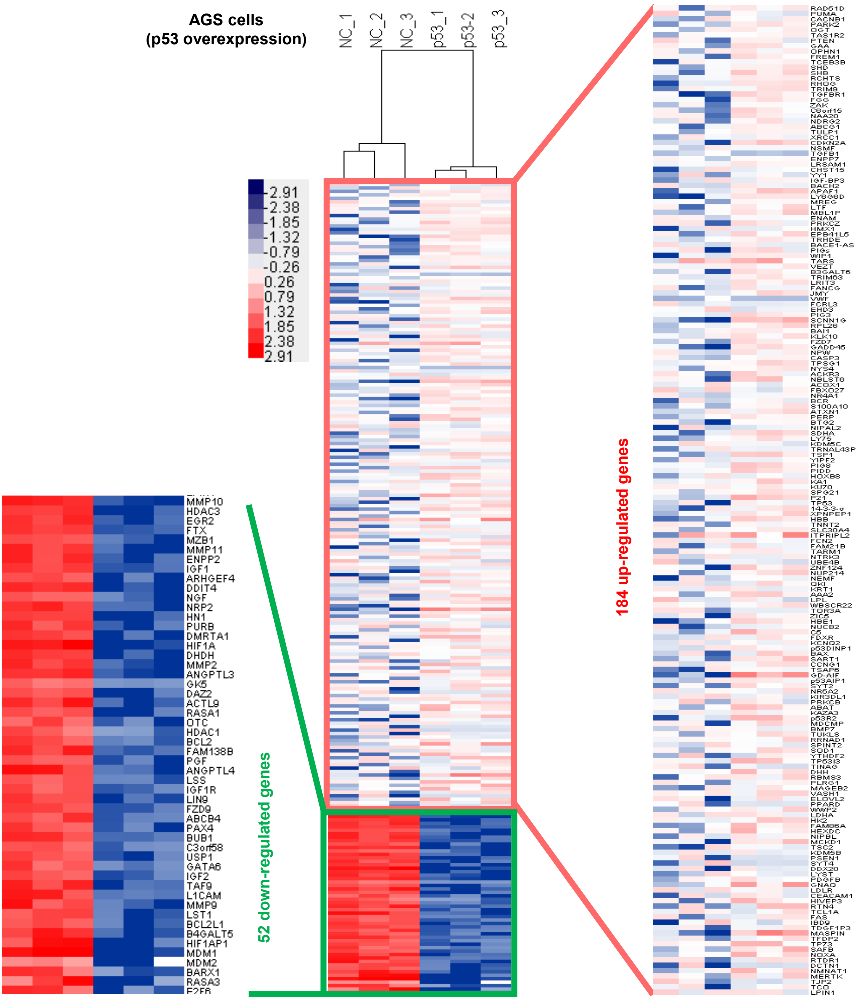

Supplementary Figure 6

A

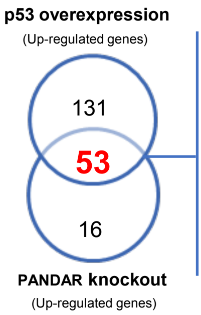

ACKR3	CEACAM1	HK2	LPIN1	NIPAL2	PIDD	RPL26	TDGF1P3	TSP1
ATXN1	CHST15	ITPRIPL2	LRSAM1	NTRK3	PIG-6	RTDR1	TINAG	VEZT
BACH2	DHH	JMY	LTF	NUP214	PLRG1	SDHA	TP53	YIPF2
C6orf15	ENPP7	KA1	MBL1P	OPHN1	PPARD	SLC30A4	TP53I3	YY1
CACNB1	GADD45	KU70	MCKD1	CDKN1A	PRKCB	SPINT2	TSAP6	ZAK
CASP3	GNAQ	LDHA	NEMF	PARK2	PRKCZ	TCO	TSC2	

B

C

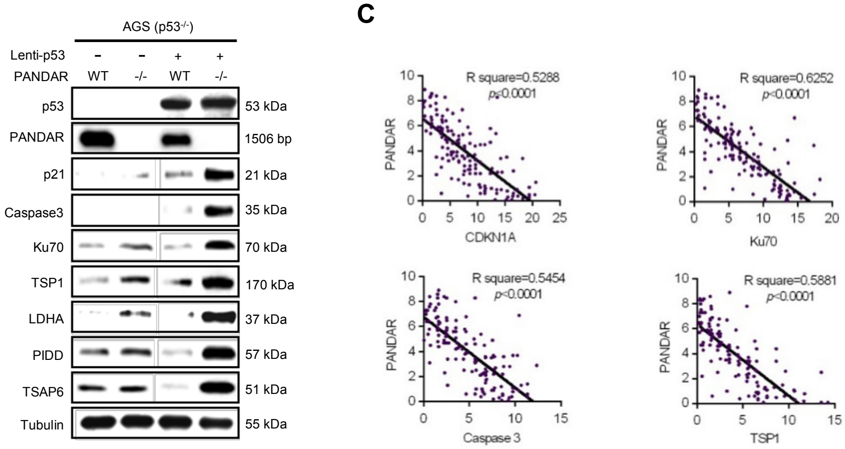

Supplementary Figure 7

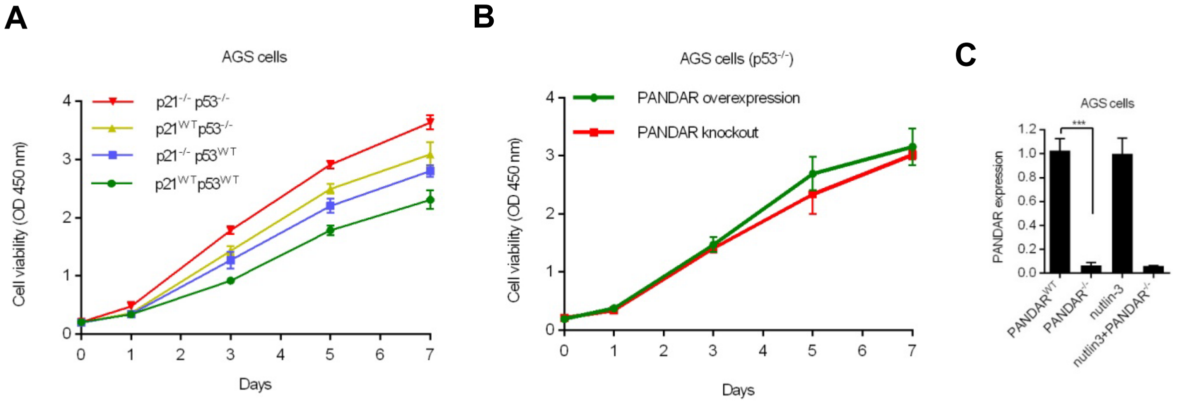

Supplementary Figure 8
